# Correction: Ferreira-Sánchez et al. Differences in Motor Imagery Ability between People with Parkinson’s Disease and Healthy Controls, and Its Relationship with Functionality, Independence and Quality of Life. *Healthcare* 2023, *11*, 2898

**DOI:** 10.3390/healthcare12212129

**Published:** 2024-10-25

**Authors:** María del Rosario Ferreira-Sánchez, Marcos Moreno-Verdú, María de los Ángeles Atín-Arratibel, Patricia Martín-Casas

**Affiliations:** 1 Department of Radiology, Rehabilitation and Physiotherapy, Faculty of Nursing, Physiotherapy and Podiatry, Complutense University of Madrid, 28015 Madrid, Spain; mrosario.ferreira@ucavila.es (M.d.R.F.-S.); matin@ucm.es (M.d.l.Á.A.-A.); pmcasas@ucm.es (P.M.-C.); 2 Department of Physiotherapy, Catholic University of Avila, 05005 Avila, Spain; 3 Department of Physical Therapy, Madrid Parkinson Association, 28011 Madrid, Spain; 4 Faculty of Experimental Sciences, Francisco de Vitoria University, 28223 Pozuelo de Alarcón, Spain; 5 Brain Injury and Movement Disorders Neurorehabilitation Group (GINDAT), Institute of Life Sciences, Francisco de Vitoria University, 28223 Pozuelo de Alarcón, Spain

In the original publication [1], María de los Ángeles Atín-Arratibel and Patricia Martín-Casas were not included as authors. Ellen Poliakoff, Zacarías Sánchez Milá, David Rodríguez Sanz, Raúl Frutos Llanes, José Manuel Barragán Casas, and Jorge Velázquez Saornil were mistakenly added as authors before. The corrected authorship, affiliations and emails updated, and Author Contributions statement appears below. The authors state that the scientific conclusions are unaffected. This correction was approved by the Academic Editor. The original publication has also been updated.
