# Supplementary material for: Differences in Motor Imagery Ability between People with Parkinson’s Disease and Healthy Controls, and Its Relationship with Functionality, Independence and Quality of Life
Source: Healthcare (Basel). 2023 Nov 3;11(21):2898. doi: 10.3390/healthcare11212898 (PMC10650523; doi:10.3390/healthcare11212898)
Supplement: Supplementary file 1 [file healthcare-11-02898-s001.zip › healthcare-2664973-supplementary.pdf]

Supplementary Table S1. Correlations between Motor Imagery vividness and functional scales in people with Parkinson's Disease (PD).

	MIQ-RS Total	MIQ-RS Visual	MIQ-RS Kinaesthetic	KVIQ-34 Total	KVIQ-34 Visual	KVIQ-34 Kinaesthetic
Disease duration	r = -0.04<br>p = 0.84	r = -0.05<br>p = 0.8	r = 0.06<br>p = 0.75	r = -0.07<br>p = 0.71	r = -0.13<br>p = 0.49	r = 0.04<br>p = 0.84
MDS-UPDRS Total	r = 0.12<br>p = 0.55	r = 0.15<br>p = 0.41	r = -0.03<br>p = 0.86	r = 0.25<br>p = 0.18	r = 0.26<br>p = 0.56	r = 0.25<br>p = 0.18
MDS-UPDRS Part I	r = -0.05<br>p = 0.78	r = 0.07<br>p = 0.71	r = -0.33<br>p = 0.07	r = -0.01<br>p = 0.95	r = 0.06<br>p = 0.76	r = -0.09<br>p = 0.65
MDS-UPDRS Part II	r = 0.11<br>p = 0.57	r = 0.11<br>p = 0.57	r = 0.04<br>p = 0.82	r = 0.19<br>p = 0.3	r = 0.19<br>p = 0.31	r = 0.25<br>p = 0.17
MDS-UPDRS Part III	r = 0.9<br>p = 0.65	r = 0.11<br>p = 0.56	r = 0.07<br>p = 0.72	r = 0.28<br>p = 0.13	r = 0.29<br>p = 0.11	r = 0.28<br>p = 0.13
MDS-UPDRS Part IV	r = -0.08<br>p = 0.66	r = -0.1<br>p = 0.61	r = -0.06<br>p = 0.77	r = -0.11<br>p = 0.55	r = -0.17<br>p = 0.36	r = 0.2<br>p = 0.9
Hoehn & Yahr scale	r = 0.11<br>p = 0.54	r = 0.12<br>p = 0.51	r = 0.13<br>p = 0.5	r = 0.17<br>p = 0.36	r = 0.18<br>p = 0.33	r = 0.15<br>p = 0.42
Schwab & England scale	r = 0.04<br>p = 0.82	r = 0.04<br>p = 0.85	r = -0.03<br>p = 0.07	r = -0.04<br>p = 0.82	r = -0.12<br>p = 0.53	r = -0.06<br>p = 0.74
Berg Balance scale	r = -0.2<br>p = 0.29	r = -0.17<br>p = 0.37	r = -0.32<br>p = 0.8	r = -0.26<br>p = 0.17	r = -0.25<br>p = 0.17	r = -0.31<br>p = 0.09
Timed Up and Go test	r = 0.13<br>p = 0.48	r = -0.13<br>p = 0.49	r = -0.01<br>p = 0.1	r = -0.09<br>p = 0.64	r = -0.02<br>p = 0.91	r = -0.01<br>p = 0.95
Box and Blocks test (dom)	r = -0.01<br>p = 0.1	r = 0.15<br>p = 0.42	r = -0.09<br>p = 0.64	r = -0.05<br>p = 0.81	r = 0.12<br>p = 0.52	r = -0.21<br>p = 0.25
Box and Blocks test (non-dom)	r = -0.02<br>p = 0.92	r = 0.03<br>p = 0.87	r = 0.01<br>p = 0.1	r = -0.06<br>p = 0.77	r = 0.6<br>p = 0.75	r = -0.16<br>p = 0.38
SF-36 Physical subscale	r = -0.16<br>p = 0.38	r = -0.67<br>p = 0.37	r = -0.13<br>p = 0.5	r = -0.14<br>p = 0.44	r = -0.21<br>p = 0.26	r = -0.16<br>p = 0.39
SF-36 Cognitive subscale	r = -0.07<br>p = 0.73	r = -0.8<br>p = 0.67	r = 0.1<br>p = 0.61	r = -0.01<br>p = 0.94	r = -0.1<br>p = 0.59	r = 0.01<br>p = 0.99

Abbreviations: KVIQ-34, Kinaesthetic and Visual Imagery Questionnaire-Extended Version; MIQ-RS, Movement Imagery Questionnaire-Revised Second Edition; SF-36, Short Form Health Survey-36; MDS-UPDRS, Movement Disorder Society - Unified Parkinson's Disease Rating Scale.

Supplementary Table S2. Correlations between Motor Imagery temporal accuracy and functional scales in people with Parkinson's Disease (PD).

	iTUG (secs)	iTUG (%)	iBBT dom (secs)	iBBT dom (%)	iBBT non-dom (secs)	iBBT non-dom (%)
Disease duration	r = 0.01<br>p = 0.96	r = -0.19<br>p = 0.31	r = 0.22<br>p = 0.25	r = 0.19<br>p = 0.32	r = -0.21<br>p = 0.25	r = -0.26<br>p = 0.17
MDS-UPDRS Total	r = -0.05<br>p = 0.8	r = -0.2<br>p = 0.27	r = 0.19<br>p = 0.3	r = 0.11<br>p = 0.57	r = 0.12<br>p = 0.53	r = 0.07<br>p = 0.72
MDS-UPDRS Part I	r = -0.32<br>p = 0.08	**r = -0.43**<br>**p = 0.02***	r = 0.12<br>p = 0.51	r = 0.04<br>p = 0.84	r = 0.04<br>p = 0.83	r = -0.04<br>p = 0.83
MDS-UPDRS Part II	r = 0.08<br>p = 0.65	r = 0.28<br>p = 0.13	r = 0.31<br>p = 0.09	r = 0.22<br>p = 0.24	r = 0.17<br>p = 0.37	r = 0.12<br>p = 0.5
MDS-UPDRS Part III	r = 0.33<br>p = 0.07	r = 0.19<br>p = 0.3	r = 0.09<br>p = 0.63	r = 0.06<br>p = 0.76	r = 0.1<br>p = 0.58	r = 0.07<br>p = 0.7
MDS-UPDRS Part IV	r = -0.07<br>p = 0.72	r = -0.11<br>p = 0.56	r = 0.17<br>p = 0.37	r = 0.11<br>p = 0.55	r = 0.09<br>p = 0.61	r = 0.07<br>p = 0.73
Hoehn & Yahr scale	r = 0.77<br>p = 0.69	r = -0.03<br>p = 0.87	r = 0.37<br>p = 0.58	r = 0.34<br>p = 0.06	r = 0.21<br>p = 0.25	r = 0.18<br>p = 0.35
Schwab & England scale	r = -0.25<br>p = 0.18	r = -0.11<br>p = 0.56	r = -0.08<br>p = 0.66	r = 0.05<br>p = 0.79	r = 0.05<br>p = 0.78	r = 0.1<br>p = 0.61
Berg Balance scale	**r = -0.43**<br>**p = 0.02***	r = -0.28<br>p = 0.13	r = 0.3<br>p = 0.1	r = -0.31<br>p = 0.09	r = -0.18<br>p = 0.33	r = -0.17<br>p = 0.36
Timed Up and Go test	**r = 0.44**<br>**p = 0.01***	r = -0.06<br>p = 0.74	r = 0.18<br>p = 0.32	r = 0.1<br>p = 0.58	r = 0.05<br>p = 0.76	r = -0.05<br>p = 0.77
Box and Blocks test (dom)	r = -0.25<br>p = 0.18	r = -0.23<br>p = 0.21	r = -0.31<br>p = 0.09	r = -0.2<br>p = 0.29	r = -0.19<br>p = 0.32	r = -0.11<br>p = 0.56
Box and Blocks test (non-dom)	r = -0.05<br>p = 0.8	r = 0.02<br>p = 0.9	r = -0.17<br>p = 0.36	r = -0.02<br>p = 0.92	r = -0.06<br>p = 0.74	r = 0.04<br>p = 0.83
SF-36 Physical subscale	r = -0.08<br>p = 0.67	r = 0.11<br>p = 0.56	r = -0.19<br>p = 0.3	r = -0.08<br>p = 0.66	r = -0.15<br>p = 0.43	r = -0.12<br>p = 0.51
SF-36 Cognitive subscale	r = 0.01<br>p = 0.97	r = 0.17<br>p = 0.73	r = 0.01<br>p = 0.95	r = 0.11<br>p = 0.54	r = -0.08<br>p = 0.66	r = -0.01<br>p = 0.96

Abbreviations: iBBT, Imagined Box and Blocks Tests; iTUG, Imagined Timed Up and Go Test; SF-36, Short Form Health Survey-36; MDS-UPDRS, Movement Disorder Society - Unified Parkinson's Disease Rating Scale. ***p<0.05**.

Supplementary Table S3. Correlations between Motor Imagery vividness and functional scales in healthy controls (HC).

	MIQ-RS Total	MIQ-RS Visual	MIQ-RS Kinaesthetic	KVIQ-34 Total	KVIQ-34 Visual	KVIQ-34 Kinaesthetic
Schwab & England scale	r = 0.31<br>p = 0.09	r = 0.31<br>p = 0.09	r = 0.31<br>p = 0.09	r = 0.31<br>p = 0.09	r = 0.3<br>p = 0.1	r = 0.31<br>p = 0.09
Berg Balance scale	r = 0.02<br>p = 0.9	r = 0.01<br>p = 0.95	r = 0.01<br>p = 0.97	r = 0.12<br>p = 0.52	r = 0.03<br>p = 0.85	r = 0.11<br>p = 0.57
Timed Up and Go test	r = -0.15<br>p = 0.42	r = -0.09<br>p = 0.64	r = -0.17<br>p = 0.35	r = -0.23<br>p = 0.21	r = -0.1<br>p = 0.6	r = -0.23<br>p = 0.21
Box and Blocks test (dom)	r = 0.06<br>p = 0.76	r = 0.01<br>p = 0.95	r = 0.08<br>p = 0.67	r = 0.01<br>p = 0.97	r = -0.15<br>p = 0.4	r = 0.05<br>p = 0.78
Box and Blocks test (non-dom)	r = 0.12<br>p = 0.53	r = 0.05<br>p = 0.8	r = 0.17<br>p = 0.35	r = 0.07<br>p = 0.7	r = -0.09<br>p = 0.64	r = 0.16<br>p = 0.39
SF-36 Physical subscale	r = 0.09<br>p = 0.65	r = 0.18<br>p = 0.33	r = 0.01<br>p = 0.97	r = 0.14<br>p = 0.44	**r = 0.36**<br>**p = 0.04***	r = 0.02<br>p = 0.91
SF-36 Cognitive subscale	r = -0.17<br>p = 0.36	r = -0.11<br>p = 0.56	r = -0.15<br>p = 0.44	r = 0.03<br>p = 0.89	r = 0.09<br>p = 0.64	r = 0.1<br>p = 0.91

Abbreviations: KVIQ-34, Kinaesthetic and Visual Imagery Questionnaire-Extended Version; MIQ-RS, Movement Imagery Questionnaire-Revised Second Edition; SF-36, Short Form Health Survey-36; UPDRS, Unified Parkinson’s Disease Rating Scale. ***p<0.05**.

Supplementary Table S4. Correlations between Motor Imagery temporal accuracy and functional scales in healthy controls (HC).

	iTUG (secs)	iTUG (%)	iBBT dom (secs)	iBBT dom (%)	iBBT non-dom (secs)	iBBT non-dom (%)
Schwab & England scale	r = -0.29<br>p = 0.12	r = -0.1<br>p = 0.59	r = -0.23<br>p = 0.23	r = -0.1<br>p = 0.59	r = -0.16<br>p = 0.38	r = -0.12<br>p = 0.51
Berg Balance scale	**r = -0.46**<br>**p < 0.01***	r = -0.2<br>p = 0.28	r = -0.03<br>p = 0.89	r = 0.18<br>p = 0.33	**r = -0.5**<br>**p < 0.01***	**r = -0.38**<br>**p = 0.04***
Timed Up and Go test	**r = 0.63**<br>**p < 0.01***	r = 0.3<br>p = 0.1	r = 0.17<br>p = 0.36	r = -0.07<br>p = 0.7	**r = 0.51**<br>**p < 0.01***	r = 0.35<br>p = 0.05
Box and Blocks test (dom)	r = -0.1<br>p = 0.59	r = 0.22<br>p = 0.24	r = 0.03<br>p = 0.9	**r = 0.42**<br>**p = 0.02***	r = -0.22<br>p = 0.23	r = 0.04<br>p = 0.82
Box and Blocks test (non-dom)	r = -0.09<br>p = 0.65	r = 0.24<br>p = 0.2	r = -0.03<br>p = 0.87	**r = 0.36**<br>**p = 0.04***	r = -0.31<br>p = 0.1	r = -0.05<br>p = 0.8
SF-36 Physical subscale	**r = -0.48**<br>**p < 0.01***	**r = -0.37**<br>**p = 0.04***	r = 0.12<br>p = 0.51	r = 0.13<br>p = 0.48	r = -0.18<br>p = 0.33	r = -0.13<br>p = 0.49
SF-36 Cognitive subscale	r = 0.02<br>p = 0.91	r = 0.11<br>p = 0.55	r = 0.2<br>p = 0.28	r = 0.22<br>p = 0.23	r = 0.06<br>p = 0.76	r = 0.08<br>p = 0.69

Abbreviations: iBBT, Imagined Box and Blocks Tests; iTUG, Imagined Timed Up and Go Test; SF-36, Short Form Health Survey-36; UPDRS, Unified Parkinson’s Disease Rating Scale. ***p<0.05**.
